# Genetic Deletion of PGF_2α_-FP Receptor Exacerbates Brain Injury Following Experimental Intracerebral Hemorrhage

**DOI:** 10.3389/fnins.2018.00556

**Published:** 2018-09-05

**Authors:** Shekher Mohan, Emily J. Koller, Jawad A. Fazal, Gabriela De Oliveria, Anna I. Pawlowicz, Sylvain Doré

**Affiliations:** ^1^Department of Pharmaceutical Sciences, Manchester University, College of Pharmacy, Natural and Health Sciences, Fort Wayne, IN, United States; ^2^Department of Anesthesiology, University of Florida, College of Medicine, Gainesville, FL, United States; ^3^Departments of Neurology, Psychiatry, Psychology, Pharmaceutics and Neuroscience, Center for Translational Research in Neurodegenerative Disease, McKnight Brain Institute, University of Florida, College of Medicine, Gainesville, FL, United States

**Keywords:** astrogliosis, hemorrhagic stroke, microgliosis, neuroinflammation, neuroprotection, prostaglandins, prostanoids

## Abstract

**Background:** The release of inflammatory molecules such as prostaglandins (e.g., PGF_2α_) is associated with brain damage following an intracerebral hemorrhagic (ICH) stroke; however, the role of PGF_2α_ and its cognate FP receptor in ICH remains unclear. This study focused on investigating the role of the FP receptor as a target for novel neuroprotective drugs in a preclinical model of ICH, aiming to investigate the contribution of the PGF_2α_-FP axis in modulating functional recovery and anatomical outcomes following ICH.

**Results:** Neurological deficit scores in FP^−/−^ mice were significantly higher compared to WT mice 72 h after ICH (6.1 ± 0.7 vs. 3.1 ± 0.8; *P* < 0.05). Assessing motor skills, the total time mice stayed on the rotating rod was significantly less in FP^−/−^mice compared to WT mice 24 h after ICH (27.0 ± 7.5 vs. 52.4 ± 11.2 s; *P* < 0.05). Using grip strength to quantify forepaw strength, results showed that the FP^−/−^ mice had significantly less strength compared to WT mice 72 h after ICH (96.4 ± 17.0 vs. 129.6 ± 5.9 g; *P* < 0.01). In addition to the behavioral outcomes, histopathological measurements were made. In Cresyl violet stained brain sections, the FP^−/−^ mice showed a significantly larger lesion volume compared to the WT (15.0 ± 2.2 vs. 3.2 ± 1.7 mm^3^; *P* < 0.05 mice.) To estimate the presence of ferric iron in the peri-hematoma area, Perls' staining was performed, which revealed that FP^−/−^ mice had significantly greater staining than the WT mice (186.3 ± 34.4% vs. 86.9 ± 13.0% total positive pixel counts, *P* < 0.05). Immunoreactivity experiments on brain sections from FP^−/−^ and WT mice post-ICH were performed to monitor changes in microgliosis and astrogliosis using antibodies against Iba1 and GFAP respectively. These experiments showed that FP^−/−^ mice had a trend toward greater astrogliosis than WT mice post-ICH.

**Conclusion:** We showed that deletion of the PGF_2α_ FP receptor exacerbates behavioral impairments and increases lesion volumes following ICH compared to WT-matched controls.Detailed mechanisms responsible for these novel results are actively being pursued.

## Introduction

Each year, approximately 795,000 Americans suffer a stroke, of which approximately 13% are attributed to intracerebral hemorrhagic (ICH) stroke (Go et al., 2014). Other than clinical management of the patient with surgical and supportive methods, there are no effective therapies for the treatment of ICH. New methods of treatment for post-stroke patients are essential to improving outcomes following a hemorrhagic brain injury. An ICH stroke is caused by the rupture of a blood vessel within the brain. While the primary injury is caused by the mass effect, a secondary injury is caused by the components of blood that make up the hematoma. For example, edema, and inflammation are major clinical concerns that are mediated by erythrocyte lysis and the release of hemoglobin and heme. Blood components released from the breakdown of the hematoma (e.g., hemoglobin, heme, and iron) result in irreversible neuronal cell death and neurological deficits (Xi et al., 2006). Following ICH, neuronal survival can be affected by changes in phenotype and function of microglia following trauma and the release of blood breakdown products. Understanding the physiopathology involved during the secondary injury of an ICH may provide further insight into inflammatory pathways and therefore potential novel targets for therapeutics.

Microglia, or infiltrating neutrophils, travel to the site of the stroke and engulf blood components and cell debris (Zhao et al., 2007). Part of this repair response involves the secretion of prostaglandins. Prostaglandins are signaling molecules that are generated and released upon cell damage and are involved in the inflammatory cascade (Minghetti et al., 1997). In the aftermath of a stroke, some prostaglandins and their receptors can be neuroprotective, while others can contribute to the injury. For example, using the mouse model of ICH stroke, our lab has shown that activation of the PGE_2_ EP1 receptor can protect against neurotoxicity and when deleted, the same receptor can exacerbate neurological outcomes following ICH (Singh et al., 2013). Also, using the same mouse model of ICH, our lab has shown that EP2^−/−^ and EP3^−/−^ mice have less ICH-induced injury compared to WT control mice (Leclerc et al., 2015b,c).

Despite the abundance of arachidonic acid in the brain, the function of its metabolite, PGF_2α_, is poorly understood. However, PGF_2α_ is known to play a significant role in the initiation of parturition, renal function, control of cerebral blood flow, and intraocular pressure principally by an increase in uveoscleral outflow of aqueous humor, autoregulation in newborn piglets, contraction of arteries, and myocardial dysfunction. Pathological conditions in humans influence PGF_2α_ levels in cerebrospinal fluid, where elevated levels of PGF_2α_ were measured following epilepsy, meningtitis, brain injury, and stroke. The FP receptor is another G-protein coupled receptor that binds selectively to PGF_2α_, which is originally synthesized from arachidonic acid (Sugimoto et al., 1994, 1997). Cyclooxygenase enzymes control the rate of transformation of arachidonic acid into the prostaglandin PGH_2_, which can then be converted by prostaglandin synthases into PGF_2α_ and other prostanoids such as PGE_2_, PGD_2_, PGI_2_, and TxA_2_ (Doré, 2006). Activation of the FP receptor triggers G_α_q protein-coupled mechanisms involving Ca^2+^ signaling, IP3 turnover and activation of protein kinase C (Toh et al., 1995). This FP receptor-mediated increase in levels of Ca^2+^ may have accounted for the increased levels of brain injury and excitotoxicity measured by our group using in a mouse model of ischemic stroke (Saleem et al., 2009; Kim et al., 2012). However, until recently, our group found that deleting, the FP receptor may also attenuate brain injury as found in a mouse model of traumatic brain injury (Glushakov et al., 2013). Currently, the role of the FP receptor in hemorrhagic stroke is undetermined and thus our goal is to elucidate the role of the FP receptor in a preclinical model of intracerebral hemorrhagic stroke.

## Methods

### Animals

Studies were performed on 2–4-month-old male adult WT (24–29 g) and FP receptor knockout (FP^−/−^) (15-21 g) C57BL/6 mice. The FP^−/−^ mice developed normally, gained weight at a rate equal to that of the WT mice and had no gross anatomical or behavioral abnormalities when compared to the WT littermates (Glushakov et al., 2013). Prior to all experiment's, PCR-genotyping was performed on all littermates and the WT mice were separated from the FP^−/−^ mice. All animal protocols were approved by the Institutional Animal Care and Use Committee of University of Florida and conducted in accordance with guidelines established by the National Institutes of Health. All mice were bred, maintained, and housed in the university's vivarium under controlled conditions (23 ± 2°C; 12 h reverse light/dark cycle), with access to food and water *ad libitum*.

### Collagenase-induced ICH model

ICH was induced in age matched male WT and FP^−/−^ mice using collagenase VII-S (0.04 units in 0.4 μL saline) (Cat. No. 9001-12-1, Sigma-Aldrich, St. Louis, MO). All mice were anesthetized with isoflurane (4% initial and 2% maintenance) and immobilized on a stereotaxic frame. A single unilateral intrastriatal injection of collagenase was given at the following coordinates relative to the bregma: 0.4 mm anterior, 2.4 mm lateral, and 3.4 mm from the dura in both WT and FP^−/−^ mice (Wang et al., 2006). Collagenase was infused at 0.2 μL/min using a stereotaxic automated injector (Stoelting, Wood Dale, IL). The needle was left in place for 10 min and then slowly removed over a 15-min period. Rectal temperature was monitored and maintained at 37.0 ± 0.5°C using a homeothermic blanket system to prevent hypothermia throughout the surgery. After the surgical procedure, the incision was closed with low toxic tissue adhesive, 3M™ Vetbond (Fischer Scientific, Pittsburgh, PA) and each mouse received a 1 mL intraperitoneal injection of warm saline to prevent dehydration. All mice were then transferred to incubators with a temperature maintained at 37.0 ± 0.5°C for recovery and monitored for 2–4 h and survived for 72 h post-ICH injury.

### Evaluation of neurological functional outcomes

Neurological functions were assessed daily post ICH (24–72 h) in the following order: neurological deficit scores (NDS), grip strength test, and accelerating rotarod test. All assessments were performed during the dark cycle (awake phase) by investigators blinded to the genotype, and for consistency, tests were performed in the same morning period of each day post-ICH. NDS scoring was measured using the 24-point scale (Clark et al., 1998). Briefly, this NDS assessment includes six individual tests (body symmetry, gait, climbing, circling behavior, front limb symmetry, and compulsory circling) scored from 0, indicating normal performance, up to 4 points on a basis of increasing severity. The sum of the scores from individual tests was reported as the NDS. The accelerating rotarod test was used to assess motor deficits using the Rota Rod Rotamex 5 machine and software (Columbus Instruments International, Columbus, OH) following ICH injury (Jones and Roberts, 1968). The rotarod tests motor deficits and coordination and is comprised of a rotating barrel, which accelerates from 4 to 30 revolutions/min over the course of 5 min. The time in seconds at which each animal fell from the barrel was recorded from a single trial using the provided software. Prior to surgery, mice were trained once daily over the course of 3 days, the average of these training periods then serving as the baseline. The grip strength test was used to assess forelimb strength by measurement using the Animal Grip Strength System (San Diego Instruments, San Diego, CA). Each mouse was placed over a steel grid by the tail so that its forelimbs were allowed to grip a single steel bar before being gently pulled backwards (away) from the bar by the tail until the grip was released. Each mouse had five consecutive trials with a 1-min rest period between trials. The data was reported as the average value of maximal force recorded before the mouse released the bar.

### Hemoglobin levels

The hemoglobin content of each brain subjected to ICH was quantified with Drabkin's reagent (Sigma-Aldrich), as described previously (Choudhri et al., 1997; Wang and Doré, 2007a). Briefly, mice were anesthetized 5 h after ICH, and transcardially perfused with 30 mL of normal saline. The brain was dissected into ipsilateral and contralateral sides, treated individually as follows. Each sample was homogenized for 2 min in 1 mL of distilled water and then centrifuged at 13,000 × g for 30 min. Eighty microliters of Drabkin's reagent was added to a 20 μL aliquot of supernatant (which contained hemoglobin) and allowed to stand for 15 min at room temperature. The concentration of cyanomethemoglobin produced was measured at 540 nm. A standard curve, reflecting the amount of hemoglobin present was generated by adding incremental volumes of blood (0, 0.5, 1.0, 2.0, 4.0, and 8.0 μL), obtained by cardiac puncture of anesthetized control mice, to 100 μL of lysate from the tissue of normal caudate putamen. Results from at least three samples per mouse were averaged.

### Immunohistology

All mice were euthanized 72 h post ICH and transcardially perfused with 4% paraformaldehyde in phosphate-buffered saline (PBS). Brains were harvested, post-fixed in perfusion solution for 48 h., and then cryopreserved in 30% sucrose/PBS solution for up to 3 days. All brains were sectioned using the Leica CM 1850 cryostat and mounted onto slides to make 10 sets of 16 sections, each 30 μM thick, equally distributed through the entire brain. Slides were then stored at −80°C until processed for histological analysis. Cresyl violet staining was used to measure corticostriatal lesion volumes. Perls' staining was used to assess the perihematoma amounts of ferric iron (Hill and Switzer, 1984). A Perls' reaction is produced through the addition of an 2% potassium ferrocyanide, which combines with Fe^3+^ to form ferric ferrocyanide, producing a bright blue color. Slides were then counterstained with nuclear fast red. Microglia and astrocyte involvement in ICH injury were studied by immunostaining using polyclonal rabbit anti-Iba1 (1:1000; Wako Bioproducts, Richmond, VA) and anti-GFAP (1:1000; DAKO, Carpinteria, CA) to measure microgliosis and astrogliosis respectively. Following overnight incubation with primary antibodies, sections were incubated with avidin-peroxidase-labeled biotin complex secondary antibodies (1:1000; BA-500, Vector Laboratories, Burlingame, CA) for 1 h Vectastain ABC and DAB SK-4100 kits (Vector Laboratories) were used per the manufacturer's protocol.

### Quantification analysis

All slides were scanned using the ScanScope CS (Aperio Technologies, Inc., Vista, CA) and ImageScope software (Aperio Technologies, Inc.) was used to perform quantitative analysis. Quantitative analysis of lesion volume was performed on 8 sections taken from 5 slides per mouse, allowing for an analysis that represented of the whole brain. Quantification of Perls' positive reaction was performed similarly to the method used to calculate lesion volume; however, only the number of positive counts around the lesion was measured. The ImageScope Positive Pixel Count algorithm was used for quantification after the appropriate brain regions were outlined. To perform quantitative analysis of cortical Iba1 and GFAP immunoreactivity, four to five sections from five slides per mouse were selected. Microgliosis and astrogliosis were analyzed by placing identically sized boxes of 1000 × 1000 pixels in both the ipsilateral and contralateral motor cortex. Data is presented as the relative ipsilateral to contralateral signal for signal normalization per area quantified. For each section within the cortex, an area immediately lateral to the lesion was selected for quantification and the intensity of Iba1 and GFAP immunoreactivity was evaluated by means of relative positive pixel counts. An analyst blinded to the experimental groups performed the entire quantifying procedure.

### Statistical analysis

All data is expressed as mean ± standard error of the mean and statistical difference between the two groups was analyzed using an unpaired two-tailed Student's *t*-test. For the neurological deficit scores (NDS), non-parametric data was calculated using the Mann–Whitney U test. When appropriate, statistical comparisons between multiple groups were done using one-way ANOVA followed by Turkey's multiple comparison tests. Statistical differences were considered significant if *P* < 0.05. All data was analyzed through GraphPad Prism 6.0 software (GraphPad Software Inc., La Jolla, CA).

## Results

### Deletion of the FP receptor exacerbates neurobehavioral deficits post-ICH

**Figure 1:**
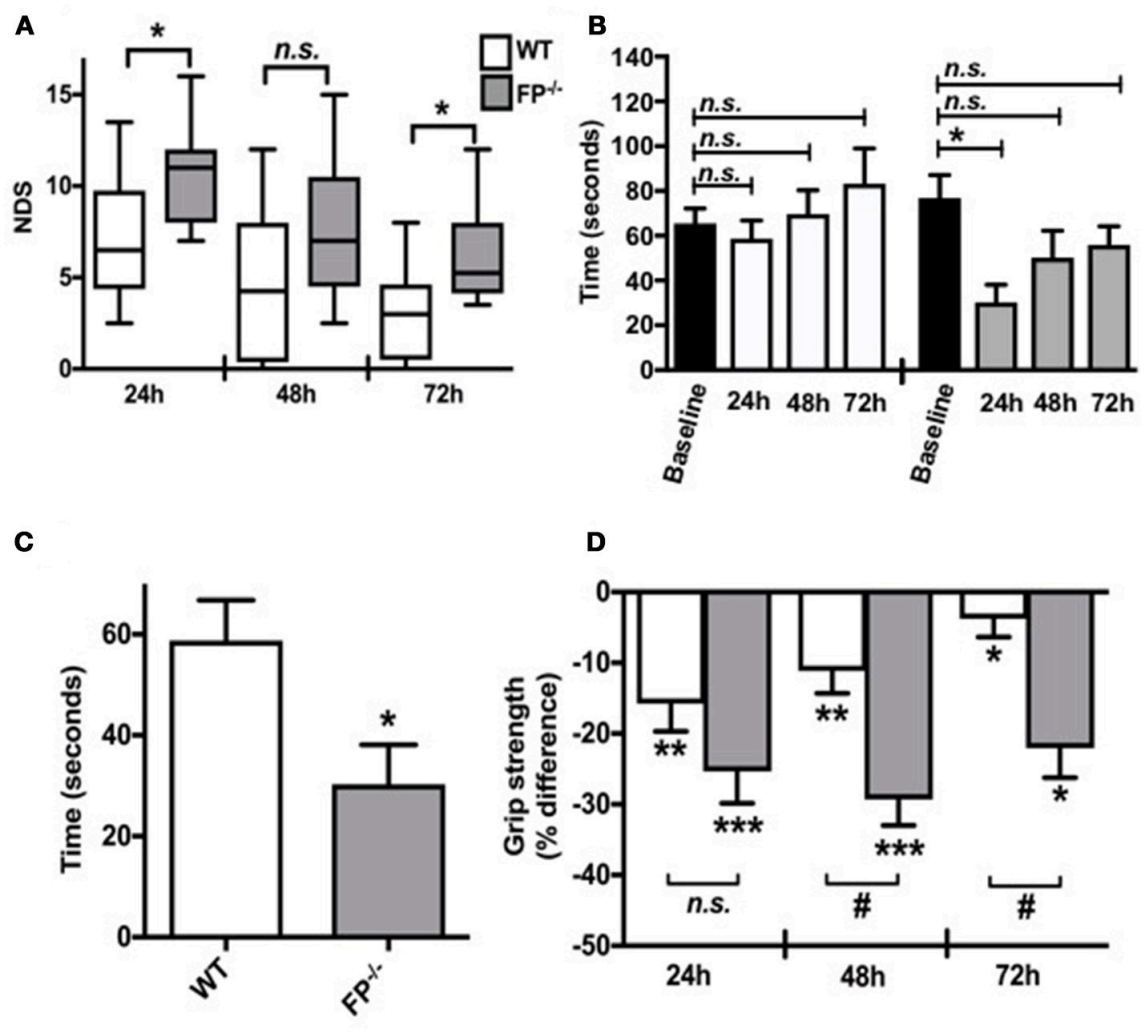
Effect of PGF_2α_-FP receptor deletion on functional neurological outcomes after ICH. Neurobehavioral testing of WT and FP^−/−^ mice was performed by investigators blinded to genotype at 24, 48, and 72 h post-ICH. **(A)** At 24 and 72 h post-ICH, FP^−/−^ mice had significantly more neurological deficit scoring compared to WT mice, and recovery was seen in both FP^−/−^ and WT mice. **(B,C)** WT and FP^−/−^ mice had similar baseline rotarod performances. Only the FP^−/−^ mice had significantly reduced latency to fall when compared to baseline function 24 h post-ICH (*P* < 0.05). The FP^−/−^ also had a greater recovery time in returning to baseline; whereas, this comparison was not obvious for the WT mice. **(D)** Changes in grip strength in FP^−/−^ and WT mice 24, 48, and 72 h post-ICH was depicted on the bar graph. Both groups showed significant changes in grip strength at all times points; however, at 48 and 72 h post-ICH FP^−/−^ mice showed significantly greater deficits in grip strength compared to the WT mice (*P* < 0.001). Both groups recovered, although the FP^−/−^ mice recovery was slower than the WT mice. All comparisons included *n* = 7–10 WT and *n* = 11 FP^−/−^ mice, and statistics were calculated using a two-way repeated measures analysis of variance with Newman–Keuls multiple comparisons test (NDS and rotarod) and a paired Student's *t*-test versus baseline (grip strength). ns = not significant, ^*^P < 0.05. ^**^P < 0.01. ^***^ and ^#^*P* < 0.001. FP = F prostanoid receptor subtype; WT = wildtype.

Neurobehavioral functional testing was performed at 24, 48, and 72 h post ICH by investigators blinded to the genotype. When compared to WT controls, the NDS of FP^−/−^ mice was significantly higher than WT mice after ICH (6.1 ± 0.7 vs. 3.1 ± 0.8; *P* < 0.05) (Figure 1A). In addition to neurological deficit analysis, rotarod and grip strength tests were performed after ICH. At 24, 48, and 72 h post ICH the FP^−/−^ mice showed reduced rotarod performances (seconds) when compared to baseline function (Figure 1B). However, only FP^−/−^ mice showed significantly reduced performance at 24 h post ICH compared to the baseline (30.3 ± 7.8 vs. 74.4 ± 15.3 s; *P* < 0.05) (Figure 1B). Additionally, when compared at 24 h post-ICH, the FP^−/−^ mice had significantly lower rotarod performance compared to WT mice (30.3 ± 7.8 vs. 58.7 ± 8.0 s; *P* < 0.05) (Figure 1C).

The grip strength test was used as an additional test to assess neuromuscular function following ICH by measuring maximal muscle strength of forelimbs. Forelimb strength was measured in five consecutive trials, with a 1-min rest between trials. Grip strength was recorded as the maximal force (in grams) and the changes at 24, 48, and 72 h post ICH were reported as the percentage (%) difference as compared to baseline (before ICH). Both FP^−/−^ and WT mice showed significant deficit with improvements in grip strength only measured in the WT mice 24, 48 and 72 h post ICH (FP^−/−^ mice: 24 h: −25.1 ± 4.6%; 48 h:−27.8 ± 4.4%; 72 h: −17.7 ± 5.0% and WT mice: 24 h:−24.3 ± 2.6%; 48 h: −16.9 ± 3.9%; 72 h: −6.8 ± 3.5%). At 48 h and 72 h post ICH, FP^−/−^ mice had greater deficits in grip strength than compared to WT mice (48 h:−27.8 ± 4.4 % vs. −16.9 ± 3.9% and 72 h: −17.7 ± 5.0 % vs. −6.8 ± 3.5 % respectively) (*P* < 0.001) (Figure 1D).

### Deletion of the FP receptor exacerbates lesion volume and increases hemoglobin level and ferric iron deposition post-ICH

**Figure 2:**
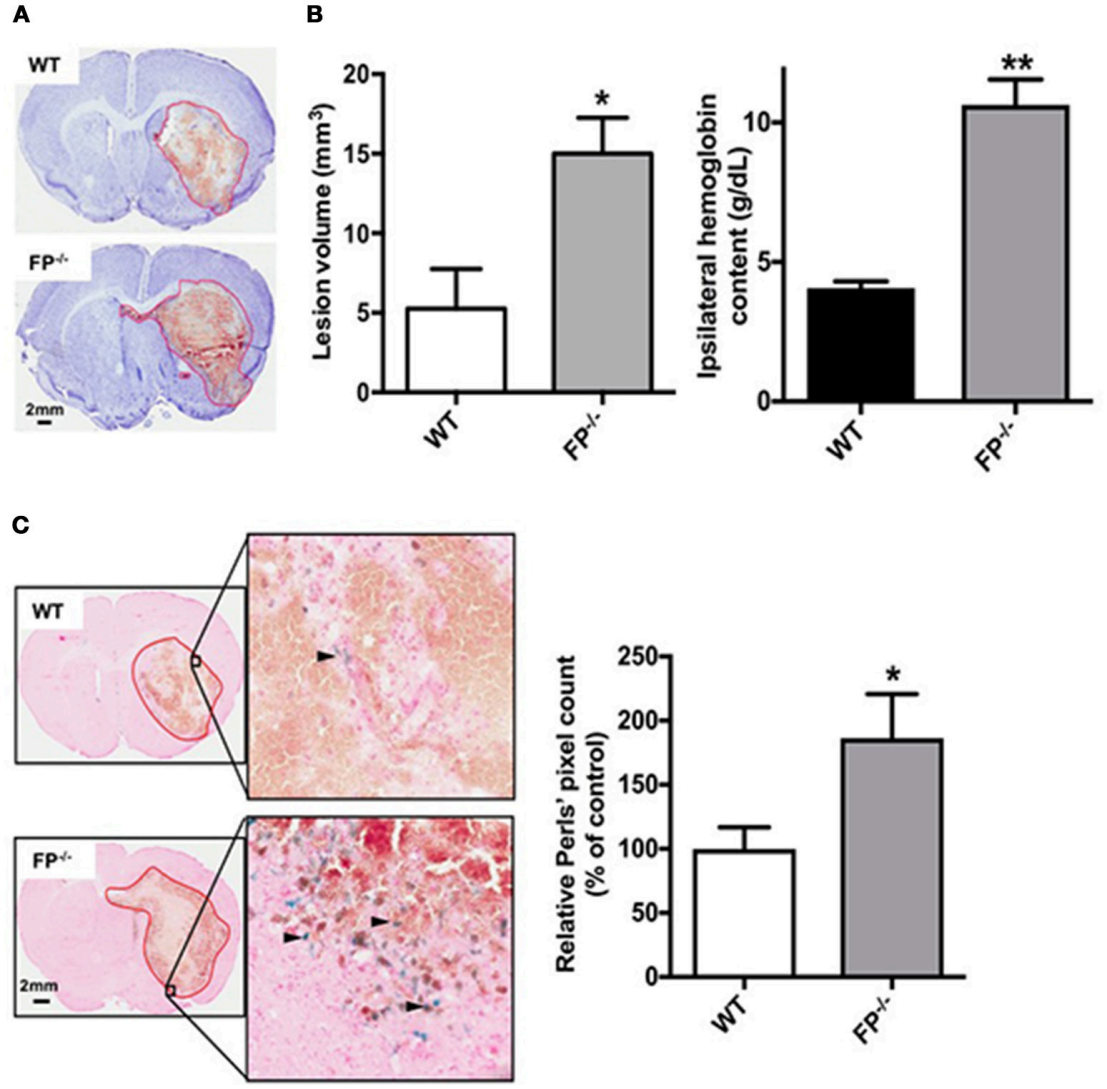
Genetic deletion of the PGF_2α_-FP receptor increases brain injury after ICH. WT and FP^−/−^ mice underwent ICH and were euthanized at 72 h for determination of lesion volume by Cresyl violet staining of brain sections. **(A)** Representative images of coronal brain sections from WT (upper panel) and FP^−/−^ mice (lower panel) show FP^−/−^ mice as having a greater lesion volume. Images were obtained from a single animal and demonstrate the characteristic hematoma profile for WT and FP^−/−^ mice, captured adjacent to the needle insertion site and representing maximal hematoma size. Quantification of lesion volumes showed that FP^−/−^ mice had significantly greater ICH-induced brain injury. (WT: *n* = 7, FP^−/−^: *n* = 10, ^*^P < 0.05). **(B)** In a separate cohort, quantification of hemoglobin content volumes at 72 h showed that FP^−/−^ mice had significantly greater ICH-induced hemoglobin content compared to WT mice (WT: *n* = 3, FP^−/−^: *n* = 3, ^**^P < 0.01). **(C)** Genetic deletion of the FP receptor increased brain ferric iron content, as represented by Perls staining post-ICH. Representative high magnification images of coronal brain sections show Perls staining (blue) in peri-hematomal regions in WT (upper panel) and FP^−/−^ mice (lower panel). Square selections in the inserts denote magnified regions. Quantification of blue positive pixel count in the ipsilateral hemisphere showed that FP^−/−^ mice had significantly greater ferric iron deposition (WT: *n* = 6, FP^−/−^: *n* = 6, ^*^P < 0.05).

Collagenase-induced ICH in mice consistently produces intrastriatal hematoma as evident from Cresyl violet staining (Figure 2A). Following analysis of the quantification, the FP^−/−^ mice had a greater lesion volume than the WT mice 72 h post ICH (15.0 ± 2.3 vs. 3.2 ± 1.7 mm^3^ respectively; *P* < 0.01).

To better understand the potential cellular mechanisms of action in FP^−/−^ mice, two additional independent measurements were taken. First, hemoglobin content was measured in WT and FP^−/−^ mice 5 h post ICH. Both the WT and FP^−/−^ mice had greater hemoglobin levels on the site-of collagenase injection (ipsilateral hemisphere). However, the FP^−/−^ mice (10.63 g/dL) had significantly more hemoglobin compared to WT mice (4.06 g/dL) (*P* < 0.01) (Figure 2B). No hemoglobin was measured on the contralateral tissue and therefore this region served as an internal control. Secondly, brain sections were assessed for the deposition of ferric iron as estimated using the Perls' staining (blue), and the levels of ferric iron were noted primarily in the perihematomal regions. Quantification of blue positive pixel count showed that FP^−/−^ mice had more ferric iron in the ipsilateral hemisphere than WT mice (186.3 ± 34.4% vs. 100.0 ± 16.8%; *P* < 0.05) (Figure 2C). Perls' positive staining was present only in or around the perihematomal region, while none was present in the contralateral hemisphere.

**Figure 3:**
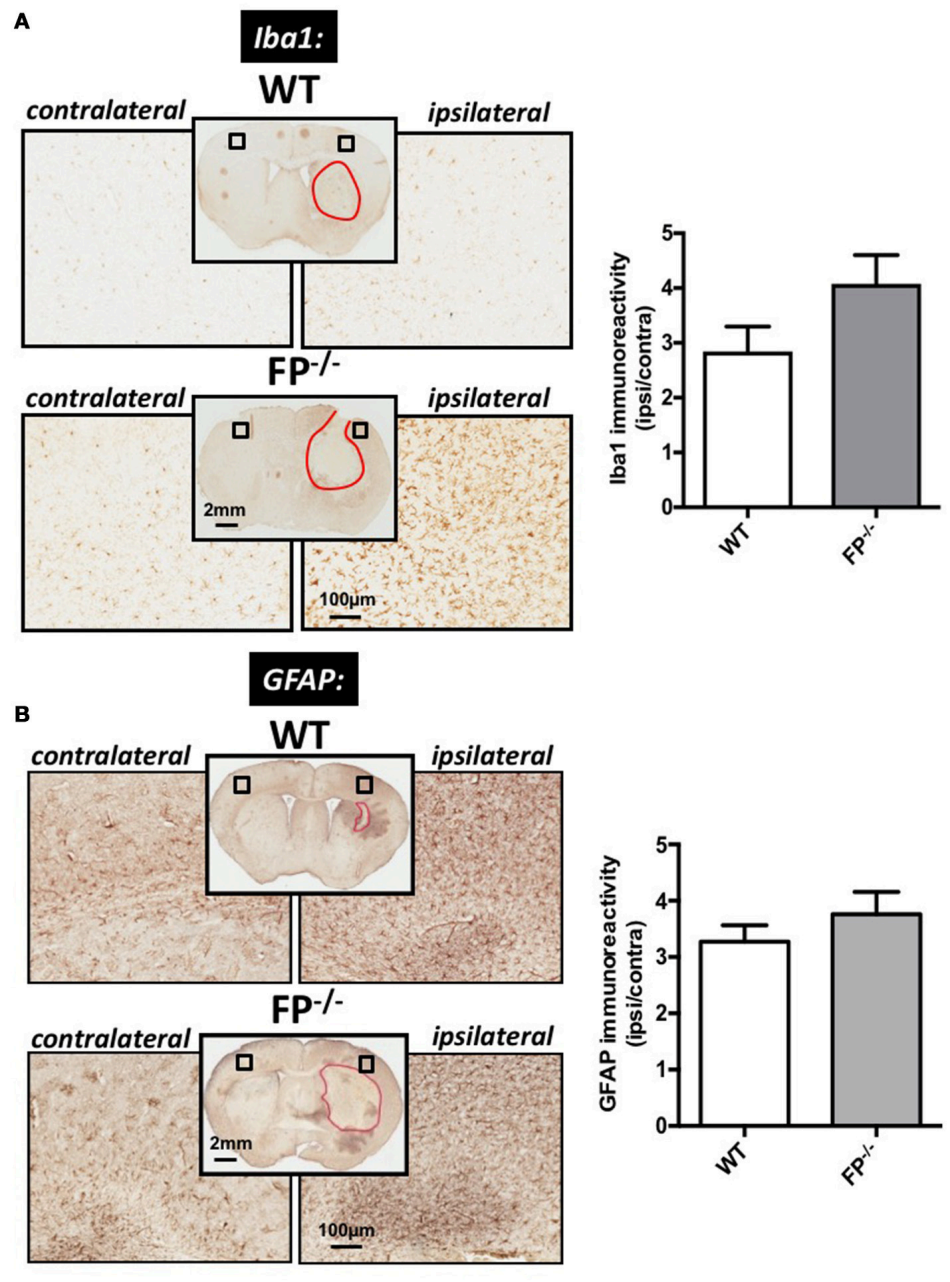
Effect of PGF_2α_-FP receptor deletion on microgliosis and astrogliosis post-ICH. At 72 h after ICH, WT, and FP^−/−^ mice were euthanized and brains processed for Iba1 and GFAP immunohistochemistry in order to evaluate cortical gliosis. **(A)** Representative high magnification images of coronal brain sections show the ipsilateral and contralateral cortex for WT (upper panels) and FP^−/−^ mice (lower panels). Square selections in the inserts denote magnified regions. Quantification of brown positive pixel count demonstrated that FP^−/−^ mice had a trend toward greater cortical Iba1 immunoreactivity then WT mice (WT: *n* = 10, FP^−/−^: *n* = 12). This trend in microglial activation was accompanied by morphological changes. **(B)** Quantification of brown positive pixel count demonstrated that FP^−/−^ mice had relatively the same level of cortical GFAP immunoreactivity as WT mice (WT: *n* = 7, FP^−/−^: *n* = 8). All data was normalized to the corresponding contralateral equivalent areas. Iba1 = Ionized calcium-binding adapter protein; GFAP = glial fibrillary acidic protein.

Microglia and astrocyte immunoreactivities were estimated in the cortical region of FP^−/−^ and WT mice brain sections using anti-Iba1 and anti-GFAP immunohistochemistry (Figure 3). ICH injury caused apparent microglia activation as detected via increased Iba1 immunoreactivity in the cortical region surrounding the lesion (area marked in red). The FP^−/−^ mice had a trend toward greater Iba1 immunoreactivity of microglia than WT mice (4.1 ± 1.8% vs. 2.8 ± 0.4%) (*P* = 0.09) (Figure 3A).

To study astrogliosis, GFAP immunoreactivity was used. Similar to the microglia marker, the GFAP levels were greater in the ipsilateral cortical region compared with the corresponding contralateral area in the ICH-treated animals. The changes in GFAP levels in the FP^−/−^ mice (3.7 ± 0.4%) were not significantly different when compared to WT mice (3.2 ± 0.3%) (Figure 3B).

## Discussion

This study investigated the role of the toxic molecule prostaglandin F_2α_ (PGF_2α_) in a mouse model of ICH stroke. The main finding of this study is that the FP^−/−^ mice have an increased susceptibility to ICH-induced injury compared to WT mice. Here, we have documented for the first time the unique and significant role of the FP receptor in exacerbating ICH injury, potentially by interfering in the functions of microglia against iron and/or heme-induced neuronal death. Our data shows that FP^−/−^ mice had significantly greater neurological deficits compared to WT controls. For example, the NDS of FP^−/−^ mice was significantly higher than WT mice after ICH (*P* < 0.05, Figure 1A). In addition to neurological deficit analysis, rotarod, and grip strength tests were performed after ICH. For the rotarod, FP^−/−^ mice showed significantly reduced performance at 24 h post-ICH compared to the baseline (*P* < 0.05, Figure 1B). The grip strength test was used as an additional test to assess neuromuscular function following ICH by measuring maximal muscle strength of forelimbs. Both FP^−/−^ and WT mice showed significant deficit in terms of improvements in grip strength 24, 48, and 72 h post ICH with the FP^−/−^ mice showing a greater deficit in grip strength compared to WT mice at the 48 and 72 h post ICH (Figure 1C).

Previous data have revealed that there is no significant difference in the morphology of cerebral vasculature and anastomoses in FP^−/−^ and WT mice brains (Glushakov et al., 2013). Therefore, the hemoglobin content 5 h post ICH between FP^−/−^ and WT mice may not be attributed to structural differences in micro-vasculature of the brain. However, greater amounts of hemoglobin following ICH might be attributed to the functions of the microvasculature. For example, the FP^−/−^ mice may have weaker blood vessel walls which may therefore be more likely to rupture under stress, such as that induced by collagenase. Also, the greater neurological deficits measured in the FP^−/−^ mice may also have been due to the greater hemoglobin content. Hemoglobin levels in the brain following hemorrhagic stroke can not only disrupt the blood brain barrier but can also up-regulate nitric oxide synthase and peroxynite formation, which would lead to further neuronal death (Ding et al., 2014). Additionally, greater expression of hemoglobin proteins (α- and β-globin) have been measured and found to be localized in neurons and microglial cells following ICH in rats (He et al., 2011). The same group also found that levels of heme and iron may cause an increase in the expression of endogenous hemoglobin after ICH.

The mouse PGF_2α_-FP receptor is reported to have the highest homology to the PGE_2_-EP1 receptor, and when activated, this receptor can increase levels of IP3 and intracellular levels of Ca^2+^ (Sugimoto et al., 1994; Mohan et al., 2012). This may explain why our findings with the FP^−/−^ mice are consistent with our previous findings with the EP1^−/−^ mice using the same ICH model; the EP1^−/−^ mice showed greater deteriorated outcomes compared to WT control mice (Singh et al., 2013). Our groups also found recently that use of EP1 receptor agonists improved anatomical outcomes and functional recovery (Leclerc et al., 2015a). In contrast to ICH, the genetic deletion and/or pharmacological blockade of either the EP1 or FP receptor types attenuated brain injury and improved neurological outcomes in excitotoxicity and mouse ischemic stroke models (Ahmad et al., 2006, 2008; Saleem et al., 2007, 2009). The difference in the role of these receptors between ischemic and hemorrhagic strokes demonstrates a uniqueness and dynamism in functionality that is determined by the type of brain injury. Differences in the vasculature might be a possible mechanism that could lead to greater lesion volumes in FP^−/−^ mice post ICH. We have previously shown that FP^−/−^ mice do not present with any significantly altered gross vascular anatomy of the brain; although we cannot rule-out changes in proteins as being responsible for the regulation of neovascularization. For example, stromal cell-derived factor 1 (SDF-1), which is controlled by endothelial cells, could alter ICH outcomes (Seo et al., 2009; Glushakov et al., 2013). These findings therefore direct our attention toward the mechanisms and cells involved in the clearance of blood in examining the etiology of increased cerebral injury in FP^−/−^ mice post ICH.

The role of the FP receptor in ischemic versus hemorrhagic stroke may be determined by the state and level of humoral neuroinflammation in intracerebral hemorrhage as compared to ischemic stroke. For example, the expression levels of pro-inflammatory cytokines IL-1β and TNFα increased early (3 h post ICH) following collagenase-induced ICH (Liesz et al., 2011). Microglia have been identified as the main producers of the early increased levels of intracerebral IL-1β and TNFα (Wang and Dore, 2007b; Wang et al., 2007). Furthermore, previous studies have investigated the activation of microglia/macrophage and leukocyte invasion after experimental ICH (Xue and Del Bigio, 2000, 2003; Loftspring et al., 2009). Nevertheless, the role of the FP receptor in microglia mediated inflammation remains largely unknown and therefore further *in vitro* studies are warranted. This study suggests that a mild activation state of microglia could be different in FP^−/−^ mice compared to WT mice post ICH. Whether changes in microglial activation significantly translate into changes in functions of the microglia remains to be explored. This study demonstrated that the FP^−/−^ mice had greater perihematomal Perls' pixel count and may support that the microglia had a reduced ability to remove ferric iron released from lysed blood cells post ICH. Previous studies have shown that microglia and astrocytes express the FP receptor and, more recently, it has been demonstrated that PGF_2α_ may enhance the clearance of β amyloid by its agonism of liver X receptors (LXR)/ retinoid X receptors (RXR) expressed on microglia (Zhuang et al., 2013). Previous evidence has shown that LXRs are expressed at high levels in the brain and when stimulated, can cause changes in the expression of inflammatory genes in microglia and macrophages (Wang et al., 2002; Joseph et al., 2003; Zelcer et al., 2007; Cui et al., 2012). To conclude, if the LXR is involved in ICH further studies would be necessary, as evidence suggests that PGF_2α_ can regulate the LXR. However, using an ischemic stroke model, activation of the LXR promoted neuroprotection and reduced inflammation via the inhibition of nuclear factor κB (Morales et al., 2008; Cheng et al., 2010). Thus, it is possible that in this study FP^−/−^ mice showed greater ICH-induced lesions with greater blood and ferric iron accumulation because of diminished phagocytic capability, resulting in decreased clearance of red blood cells and therefore increased brain injury.

In response to injury, astrocytes have a diverse role and it is well known that reactive astrocytes (gliosis) form a glial scar that contains the damaged area as shown following our ICH protocol. However, following injury and/or neurodegeneration, reactive gliosis, which involves alterations in functioning and phenotype of different glial cells, may augment brain damage. Studies have revealed that the astrocyte response to gross brain damage leads to anisomorphic (disorganized) astrogliosis that reinforces a cascade of events that eventually increases brain injury. Anisomorphic astrogliosis can inhibit neurite outgrowth and increase levels of the inducible form of nitric oxide synthase (iNOS) and nitric oxide (NO) which can possess cytotoxic properties and contribute to neuronal death (Gibbons and Dragunow, 2006). The elevated levels of NO released from activated astrocytes might be the most relevant in ICH-induced injury as NO is a vasodilator (Chi et al., 2015; Crobeddu et al., 2015; Munoz et al., 2015). Therefore, increased vasodilation could lead to greater ICH-induced secondary injury. In this study, we saw increased ipsilateral astrogliosis in both FP^−/−^ and WT mice post-ICH; however, no significance was found between the two genotype groups.

Microglia and astrocytes become activated following brain injury and release factors that contribute to the functional state of the blood-brain barrier. Here, we show that microglia may be more “reactive” than astrocytes in FP^−/−^ mice compared to WT mice. Furthermore, compared to previously published work by our group using EP1^−/−^, EP2^−/−^, and EP3^−/−^ mice post ICH, the levels of micro- and astrogliosis are contradictory compared to the FP^−/−^ mice used in this study (Singh et al., 2013; Leclerc et al., 2015b,c). The decreased level of micro- and astrogliosis in EP1^−/−^, EP2^−/−^, and EP3^−/−^ mice was used to account for the changes in functional and anatomical outcomes post ICH. We found that the FP^−/−^ mice had a trend toward greater microgliosis, suggesting that this state of activation in the FP^−/−^ mice could potentially be responsible for the changes in functional and anatomical outcomes presented here. Further *in vivo* and *in vitro* studies are necessary to elucidate the role of the FP receptor in glial cells are necessary. Proposed future experiments include performing *in vivo* experiments using the FP receptor selective antagonist and agonist to measure any FP receptor mediated-neuroprotection in this and other ICH models. Neuropharmacological experiments designed to specifically study the FP receptor will help clarify the respective role of the glial-neuronal axis after ICH.

## Conclusions

In this study, we have provided evidence that suggests a neuroprotective role for the FP receptor following ICH. Our results show that deletion of the FP receptor increases brain injury, functional deficits and increases the deposition of ferric iron post-ICH. However, without following this work with further experiments that utilize a FP receptor selective antagonist and agonist, caution should be used when interpreting the potential for the FP receptor as a therapeutic target for the treatment of ICH. Our findings are similar to those found with EP1^−/−^ mice post-ICH, and recently our group has shown that following activation of the EP1 receptor, astrogliosis, neutrophil infiltration, blood-brain barrie breakdown, and functional recovery all improved (Leclerc et al., 2015a). Based on these findings, we hypothesize that the activation of the FP receptor will result in measurable changes in the improvement of functional and anatomical outcomes following ICH. Until then, we remain hopeful that the FP receptor, similar to the related EP1 receptor, is a viable therapeutic target for the treatment of ICH.

## Author contributions

SM and SD designed the study, analyzed, and interpreted the results and wrote the manuscript. SM performed the surgical procedures, performed the blinded behavioral testing, and harvest brains for analysis with the assistance of the other lab members. EK, JF, GD, and AP coordinated and performed tissue sections and contributed to histological staining, quantification and data analysis. All authors read and approved the final manuscript.

### Conflict of interest statement

The authors declare that the research was conducted in the absence of any commercial or financial relationships that could be construed as a potential conflict of interest.
